# Colony-stimulating activity from the new metastatic TS/A cell line and its high- and low-metastatic clonal derivatives.

**DOI:** 10.1038/bjc.1985.180

**Published:** 1985-08

**Authors:** G. Nicoletti, P. Brambilla, C. De Giovanni, P. L. Lollini, B. Del Re, A. Marocchi, P. Mocarelli, G. Prodi, P. Nanni

## Abstract

We investigated the presence of colony-stimulating factor (CSF) in supernatants obtained from TS/A, a new metastatic murine cell line, and from its high-and low-metastatic clonal derivatives (E and F clones, respectively). TS/A cells produced a CSF in vitro that induced proliferation and differentiation of murine monocytic and granulocytic progenitors in agar cultures. In TS/A-bearing mice remarkable splenomegaly, blood granulocytosis and thymus depletion were observed along with a stimulatory activity in serum and a strong proliferative activity both in spleen and in bone marrow populations. Conditioned media from E clones showed an in vitro colony-stimulating activity greater than those of F clones. Mice injected subcutaneously with cells of all clones studied showed granulocytosis, splenomegaly and thymus depletion, although to varying degrees. However, no direct correlation between granulocytosis-splenomegaly and the number of spontaneous lung metastases was observed.


*Br. J. Cancer* (1985), **52**, 215–222

### Colony-stimulating activity from the new metastatic TS/A cell line and its high- and low-metastatic clonal derivatives

G. Nicoletti[1], P. Brambilla[2], C. De Giovanni[1], P.-L. Lollini[1], B. Del Re[1], A. Marocchi[2], P. Mocarelli[3], G. Prodi[1] & P. Nanni[1]

[1]*Istituto di Cancerologia, Universita' di Bologna, Viale Filopanti 22, I-40126 Bologna;* [2]*Ospedale Civile di Desio, I-20033 Desio, Milano; and* [3]*Istituto di Patologia Generale, Universita' di Milano, Italy.*

**Summary** We investigated the presence of colony-stimulating factor (CSF) in supernatants obtained from TS/A, a new metastatic murine cell line, and from its high-and low-metastatic clonal derivatives (E and F clones, respectively). TS/A cells produced a CSF *in vitro* that induced proliferation and differentiation of murine monocytic and granulocytic progenitors in agar cultures. In TS/A-bearing mice remarkable splenomegaly, blood granulocytosis and thymus depletion were observed along with a stimulatory activity in serum and a strong proliferative activity both in spleen and in bone marrow populations. Conditioned media from E clones showed an *in vitro* colony-stimulating activity greater than those of F clones. Mice injected subcutaneously with cells of all clones studied showed granulocytosis, splenomegaly and thymus depletion, although to varying degrees. However, no direct correlation between granulocytosis-splenomegaly and the number of spontaneous lung metastases was observed.

Haemopoietic alterations, with marked granulocytosis and myeloid spleen hyperplasia, with no evidence of infections, have been frequently observed in animals during the growth of transplantable tumours (Burlington *et al.*, 1977; Reincke *et al.*, 1978; Lee *et al.*, 1980; Balducci & Hardy, 1983). Granulocytosis has also been reported sometimes in patients with various cancers (Okabe *et al.*, 1978, 1982; Suda *et al.*, 1980) and in nude mice transplanted with human tumours (Asano *et al.*, 1977; Okabe *et al.*, 1978; Sato *et al.*, 1979; Suda *et al.*, 1980; Mizoguchi *et al.*, 1982; Okabe *et al.*, 1982). Moreover, colony-stimulating activity has been detected in conditioned media from cell lines derived from murine (Burlington *et al.*, 1977; Balducci & Hardy, 1983; Milas *et al.*, 1984) and human (Okabe *et al.*, 1982) transplantable tumours.

Quantitative and qualitative haemopoietic alterations could play an important role in the metastatic process and the study of the production of colony-stimulating factor(s) (CSF) by metastasizing tumours could help to clarify if and when such an influence exists. Therefore, we studied the production of colony-stimulating activity by TS/A line, a new murine spontaneous mammary carcinoma cell line that metastasizes spontaneously to the lungs (Nanni *et al.*, 1983) and by its high- and low-metastatic clonal derivatives (Lollini *et al.*, 1984). Data are presented here on the haemopoietic alterations observed during *in vivo* growth of TS/A and clones, on the detection of *in vitro* produced CSF and on the comparison between this activity and metastatic potential.

Correspondence: P. Nanni
Received 14 January 1985; and in revised form 11 April 1985.

### Materials and methods

### *Mice*

Eight-12 week-old female BALB/cAnNCR1BR mice (hereafter referred to as BALB/c), purchased from Charles River, Calco, Italy, or bred in our facilities were used throughout the study.

### *Cell lines and clonal derivatives*

The parental TS/A cell line was derived from a spontaneous mammary adenocarcinoma which arose in a retired breeder BALB/c female (Nanni *et al.*, 1983). Clonal derivatives with different metastatic potential, which have been previously selected and characterized (Lollini *et al.*, 1984), were also used in the present study: E1, E2 and E3 clones are highly metastatic and F1, F2 and F5, are poorly metastatic. In the experiments here reported, TS/A cells were between the 20th and the 35th *in vitro* passage and clones were between the 30th and the 45th *in vitro* passage after cloning.

A cell line derived from the B16 melanoma of C57BL/6 origin (kindly provided by Dr A. Mantovani, Istituto Mario Negri, Milan, Italy) was used as a control in some experiments.

All cell lines and clones were cultured in Dulbecco's MEM supplemented with 2 mM glutamine, $100\,U\,ml^{-1}$ penicillin, $100\,\mu g\,ml^{-1}$

© The Macmillan Press Ltd., 1985

streptomycin (referred to as DMEM) and with 10% heat-inactivated foetal calf serum (FCS) (GIBCO, Paisley, Scotland) in a 5% $CO_2$ humidified atmosphere at 37°C and were routinely subcultured twice weekly by trypsin-EDTA treatment.

*Assays for CSF activity*

Assays for detection of CSF production were made on conditioned media obtained as follows: $1-2 \times 10^6$ cells were seeded in $75\,cm^2$ flasks and cultured in DMEM + 10% FCS. Supernatants were collected 3 days later, centrifuged at 3000 rpm for 20 min and filtered through $0.45\,\mu m$ Millex filters (Millipore). At the same time, monolayers were processed to determine cell number/flask.

The ability of conditioned media to induce bone marrow proliferation was tested as reported elsewhere (Pessina *et al.*, 1981): $10^5$ bone marrow cells from normal BALB/c mice were cultured in 35 mm Petri dishes with 1 ml semisolid medium (McCoy's 5A medium, 20% FCS [GIBCO], 0.3% Difco agar [DIFCO Laboratories, Detroit, Michigan, USA]) supplemented with conditioned medium at 0.31–40% final concentration. After a 7-day culture, plates were scored microscopically and colonies of $\geq 50$ cells were counted. Colony morphology was evaluated after staining of the whole culture dish with May Grumwald-Giemsa solution as reported by Konwalinka *et al.* (1982).

[$^3$H]-TdR uptake assay for CSF activity was performed according to Prystowsky *et al.* (1984) with slight modifications: $5 \times 10^4$/well BALB/c normal bone marrow cells were seeded in Microtest II plates (Nunc, Denmark) in DMEM + 15% FCS in the presence of conditioned medium at 2.5–40% final concentration. After 4-day culture, cells were pulsed for 4 h with $1\,\mu Ci$ [$^3$H]-methylthymidine ([$^3$H]-TdR) ($6.7\,Ci\,mmol^{-1}$ specific activity; NEN, (Dreieich, Germany) and then processed by a cell harvester (Skatron, Norway). Radioactivity was counted in an Intertechnique SL 3000 scintillation spectrometer (Plaisir, France), provided with $^{226}Ra$ external standard.

In vivo *studies*

Mice were injected s.c. with $10^5$ or $10^6$ viable cells. During tumour growth the following parameters were examined: tumour volume (calculated as $4/3\,\pi . [(a+b)/4]^3$, where $a$ = maximal tumour diameter and $b$ = tumour diameter perpendicular to $a$), or tumour weight at sacrifice; peripheral leukocytes (no. $mm^{-3}$) and percentage of peripheral lymphocytes, spleen weight, spleen and thymus cell yield.

Spleen cells from TS/A-bearing mice were studied by cytochemical (PAS-, ANAE- and peroxidase stains) and immunofluorescence techniques: surface immunoglobulins (SIg) were visualized with anti-mouse IgG FITC-conjugated antiserum from Miles; Thy 1 antigen was detected by anti-Thy 1.2 monoclonal antiserum from NEN.

Proliferative activity of spleen and bone marrow cells from tumour-bearing mice was tested in a [$^3$H]-TdR uptake assay: $4 \times 10^5$ spleen cells or $5 \times 10^4$ bone marrow cells (collected from femurs) were plated in each well of Microtest II plates (Nunc, Denmark), incubated for 96 h, with a final 4 h pulse with $1\,\mu Ci$ [$^3$H]-TdR, and then processed as above.

Lung metastases were enumerated as previously described (Nanni *et al.*, 1983) with the aid of a dissecting microscope.

### Results

*Haemopoietic alterations in TS/A-bearing mice*

Progressive granulocytosis, splenomegaly, and thymus depletion were observed in BALB/c mice s.c. injected with the TS/A cell line (Table I). Splenomegaly was already detectable 1 week after cell injection: both spleen weight and cell yield reached values which were more than 5-fold greater than control levels by the 30th day after inoculation. At this time, spleen comprised ~10% T cells (Thy 1-positive cells), ~20% B lymphocytes (SIg positive), ~20% mature monocytes (PAS- and ANAE-positive, peroxidase-negative) and ~50% hypogranular metamyelocytes and polymorphonuclear cells (PAS- and peroxidase- partially positive, ANAE-negative). Leukocytosis reached values of $>200{,}000\,mm^{-3}$ and was mainly due to enlargement of the granulocytic population. The yield of bone marrow cells in TS/A-bearing mice seemed to be unaffected (data not shown).

A stimulating activity on the proliferation of murine bone marrow cells was detected, by means of the [$^3$H]-TdR uptake assay, in serum from 30-day TS/A-bearing mice (Table II). The stimulating activity was proportional to the number of s.c. injected TS/A cells and significantly greater than the activity of control serum.

Such a stimulating activity in the serum of TS/A-bearing mice could result in alterations in proliferative activity of spleen and bone marrow. Therefore, we evaluated both the spontaneous [$^3$H]-TdR uptake in cells from these organs at different times after TS/A s.c. injection and their susceptibility to the addition of TS/A conditioned medium. Data from 30-day TS/A-bearing mice are shown in Table III. Both spleen and bone marrow cells from tumour-bearing animals showed a higher proliferative activity than cells from controls: such a difference was already evident by 1 week after

**Table I** *In vivo* characterization of TS/A-bearing mice

| *Days after injection*[a] | *Tumour weight (mg)* | *Spleen weight (mg)* | *Leukocytes* $\times 10^3$ $mm^{-3}$ | *Lymphocytes* $\times 10^3$ $mm^{-3}$ | *% of lymphocytes* | *Thymus cell yield* ($\times 10^6$) |
|---|---|---|---|---|---|---|
| 0 | — | 93±6 (n=10) | 8.7±1.4 (n=10) | 6.7±1.1 (n=10) | 77±3 (n=10) | 94±19 (n=4) |
| 7 | 330 | 192 | 8.7 | 5.4 | 62 | 54 |
| | 337 | 133 | 8.4 | 4.4 | 52 | 44 |
| 14 | 490 | 162 | 7.2 | 3.1 | 43 | 24 |
| | 602 | 151 | 5.7 | 2.6 | 45 | 24 |
| 21 | 1803 | 289 | 19.5 | 3.7 | 19 | ND |
| | 1735 | 324 | 21.7 | 3.3 | 15 | ND |
| 28 | 2631 | 534 | 38.9 | 4.3 | 11 | 4 |
| | 2190 | 591 | 50.4 | 4.0 | 8 | 9 |
| 35 | ND | 764 | 134.0 | 8.0 | 6 | ND |
| | ND | 491 | 225.5 | 15.8 | 7 | ND |

[a]$10^6$ TS/A cells were injected s.c. in 10 female BALB/c mice; each week two animals were killed.
ND = not determined.

**Table II** Stimulation of [$^3$H]-TdR uptake in normal bone marrow cells by serum of TS/A-bearing BALB/c mice[a]

| *Control uptake*[b] | [$^3$*H*]-*TdR uptake*[b] *in the presence of BALB/c serum* | | | |
|---|---|---|---|---|
| | | *% serum* | | |
| | *BALB/c treatment* | *2.5* | *5* | *10* |
| 277 ±34 | None | 1,278 ±120 | 1,344 ±119 | 2,305 ±142 |
| | $10^5$ TS/A cells | 1,789 ±139 | 3,308 ±221 | 4,817 ±167 |
| | $10^6$ TS/A cells | 3,158 ±315 | 9,729 ±426 | 16,280 ±1316 |

[a]30 days after TS/A cells injection.
[b]Mean d.p.m. ± s.e. from six replicates.

**Table III** [$^3$H]-TdR uptake in spleen and in bone marrow cells obtained from normal and TS/A-bearing[a] BALB/c mice and cultured in the absence and in the presence of TS/A conditioned medium

| *Cells* | *Source* | [$^3$*H*]-*TdR uptake*[b] *Control* | *+10% TS/A conditioned medium* |
|---|---|---|---|
| Spleen | Normal BALB/c | 2,440±207 | 5,584±724 |
| | TS/A-bearing BALB/c[a] | 28,956±1989 | 100,791±6,549 |
| Bone marrow | Normal BALB/c | 163±13 | 215,736±10,511 |
| | TS/A-bearing BALB/c[a] | 1,544±177 | 274,801±12,278 |

[a]30 days after $10^6$ TS/A cells injection.
[b]Mean d.p.m. ± s.e. from six replicates.

TS/A injection (data not shown). Moreover, when cells were cultured in the presence of 10% TS/A conditioned medium, spleen cells from TS/A-bearing mice were further stimulated to proliferate reaching values of [$^3$H]-TdR uptake much higher than those of control cells; bone marrow cells from normal and tumour-bearing animals also showed high and similar levels of [$^3$H]-TdR uptake.

### *CSF in TS/A conditioned medium*

To investigate whether the TS/A cell line could directly interfere with haemopoiesis by production of CSF(s), and whether it retains this production *in vitro*, supernatants from TS/A cultures were collected and tested for their ability to induce bone marrow proliferation in agar cultures and in microwell cultures. TS/A conditioned medium was able to induce proliferation of normal murine bone marrow cells in agar cultures, in which granulocyte-macrophage colonies were observed (Figure 1a). A strong stimulating activity on murine bone marrow cells was also detected by means of the [$^3$H]-TdR uptake assay performed in the presence of TS/A conditioned medium: data from a representative experiment are given in Figure 1b, where up to a 2,000-fold stimulation was obtained. Conditioned medium from an unrelated cell line (B16 melanoma) showed no activity (data not shown).

### *Colony-stimulating activity in TS/A clonal derivatives*

We had recently derived high- and low-metastatic clones (E and F clones, respectively) from the parental TS/A cell line (Lollini *et al.*, 1984). To investigate whether all clonal derivatives produced CSF and whether a direct relationship occurred between such an activity and metastatic potential, we compared three high-metastatic E clones with three low-metastatic F clones.

Conditioned media from all 6 clones were able to

**Figure 1** Effect of TS/A conditioned medium on *in vitro* cultures of normal BALB/c bone marrow cells: (a) induction of granulocyte-macrophage colonies in agar cultures (difference between replicates was always <5%). Control = 0 colonies. (b) stimulation of [$^3$H]-TdR uptake (standard error did not exceed 10% of the mean of 6 replicate values). Control = 163 dpm.

induce proliferation of murine monocytic and granulocytic progenitors in agar cultures yielding pure granulocyte, macrophage and mixed colonies (Figure 2a), but supernatants from E clones induced higher numbers of colonies than supernatants from F clones. A higher *in vitro* activity by conditioned media from E clones was also shown by means of [$^3$H]-TdR uptake in murine bone marrow cells (Figure 2b). In both assays supernatants from F clones also showed an activity lower than those observed with E clones when the plateau was reached (at concentrations of conditioned medium ranging from 10 to 40% and from 20 to 40% for the colony and the [$^3$H]-TdR uptake assays, respectively). The differences among clones in cell size and in doubling time cannot account for this phenomenon.

In all clones examined, tumour-bearing mice showed progressive leukocytosis, mainly due to enlargement of the granulocytic population, even though values of leukocytes among different clones were scattered (Table IV). On the whole, comparison between E and F clones did not show different group patterns. It should be underlined that a very low leukocytosis was observed in F5 tumour-bearing mice. Animals were then sacrificed when individual mean tumour diameters exceeded 2.2 cm, and leukocytes no. mm$^{-3}$, spleen weight and number of lung metastases were evaluated for each group (Table V). Leukocytosis and splenomegaly did not appear to be directly related to the number of lung metastases: the F2 clone induced the highest leukocytosis and splenomegaly but a very low number of metastases. It is emphasized that, even when comparison among clones was made in conditions of similar tumour dimensions, the leukocytosis induced by the F5 clone (near 24,000 mm$^{-3}$) remained much lower than that observed with other clones.

### Discussion

Qualitative and quantitative haemopoietic alterations could play an important role in the metastatic process. Enhancement of lung colonization has been reported to occur in association with increasing granulocytosis (Milas *et*

**Figure 2** Effect of conditioned media from TS/A clones on *in vitro* cultures of normal BALB/c bone marrow cells: (a) induction of granulocyte-macrophage colonies in agur cultures (difference between replicates was always <5%). Control = 0 colonies; (b) stimulation of [$^3$H]-TdR uptake (standard error did not exceed 10% of the mean of 6 replicate values). Control = 293 dpm. (■) E1; (●) E2; (▲) E3; (○) F1; (△) F2; (□) F5.

**Table IV** Haematological parameters of BALB/c mice during *in vivo* growth of TS/A clones

| | *Days after s.c. injection of $10^5$ cells* | | | | | |
|---|---|---|---|---|---|---|
| | *15* | | | *35* | | |
| *Clone*[a] | *Tumour volume*[b] | *Leukocytes* $\times 10^3 mm^{-3}$ | *% of lymphocytes* | *Tumour volume*[b] | *Leukocytes* $\times 10^3 mm^{-3}$ | *% of lymphocytes* |
| E1 | 1.0±0.2 | 10.4±1.2 | 35±3 | 8.2±0.6 | 97.8±23.0 | 6±1 |
| E2 | 0.3±0.1 | 7.5±1.1 | 48±8 | 4.7±0.6 | 47.1±12.2 | 18±6 |
| E3 | 0.5±0.1 | 11.3±1.6 | 37±5 | 5.8±0.7 | 101.9±21.6 | 7±1 |
| F1 | 0.2±0.1 | 6.2±0.8 | 64±5 | 2.4±0.5 | 73.4±39.9 | 16±6 |
| F2 | 0.7±0.2 | 10.7±1.0 | 34±3 | 6.5±0.8 | 350.5±25.7 | 3±1 |
| F5 | 0.1±0.0 | 5.7±0.9 | 79±4 | 2.1±0.3 | 11.6± 1.5 | 33±3 |

[a]5 animals/group.
[b]See **Materials and methods.**

**Table V** Leukocytosis, splenomegaly and spontaneous lung metastases in BALB/c mice injected s.c. with TS/A clones and sacrificed when tumour diameter exceeded 2.2 cm

| *Clone*[a] | *Tumour volume*[b] | *Time at sacrifice*[c] | *Leukocytes* $\times 10^3 mm^{-3}$ | *Spleen weight (mg)* | *Median no. of lung metastases* |
|---|---|---|---|---|---|
| E1 | 7.4±0.7 | 37±1 | 100.3±14.7 | 522±40 | >200 |
| E2 | 7.6±0.5 | 43±2 | 115.5±24.1 | 555±57 | 101 |
| E3 | 6.1±0.4 | 39±1 | 119.0±18.7 | 550±33 | >200 |
| F1 | 6.1±0.2 | 47±3 | 161.1±31.6 | 450±50 | 36 |
| F2 | 6.6±0.7 | 37±1 | 375.4±22.6 | 1,119±61 | 16 |
| F5 | 7.7±0.6 | 57±4 | 24.0± 3.9 | 357±17 | 14 |

[a]5 animals/group.
[b]See **Materials and methods.**
[c]Days after cell injection.

*al.*, 1984) and a correlation between splenomegaly and metastases has been suggested (Sato *et al.*, 1981).

We studied *in vitro* colony-stimulating activity and *in vivo* haemopoietic alterations in the new murine TS/A cell line, that has been derived from a spontaneous mammary carcinoma and is able to metastasize spontaneously to the lung (Nanni *et al.*, 1983). Moreover, we examined two sets of clones selected from TS/A line, which are all able to metastasize spontaneously but strongly differ in metastatic potential (E clones induce higher numbers of lung metastases than F clones) (Lollini *et al.*, 1984).

TS/A conditioned medium exerted a colony-stimulating activity on granulocyte-macrophage progenitors. Moreover, when the TS/A line was injected s.c., progressive spleen enlargement and thymic atrophy as well as a dramatic increase in peripheral granulocytic population were observed. Even though the TS/A line is able to metastasize, spleen was free from metastases and not able to induce tumours, when injected into syngeneic animals (data not shown). Serum from TS/A-bearing mice was also found to stimulate proliferation of normal murine bone marrow cells. Therefore, a colony-stimulating activity was detected both in TS/A conditioned medium and in serum from TS/A bearing animals. However, the possibility that the alterations we observed *in vivo* could be due also to an interaction between tumour and host cells cannot be ruled out.

*In vitro* production of CSF and *in vivo* occurrence of splenomegaly and granulocytosis have been shown for all the TS/A clonal derivatives examined. We observed a discrepancy between *in vitro* and *in vivo* assays. *In vitro* both agar colony and [$^3$H]-TdR uptake assays seem to indicate that supernatants from E clones have a CSF activity higher than those of F clones. On the contrary, such a pattern was not revealed by *in vivo* studies: mice injected with F2 cells displayed the strongest leukocytosis and splenomegaly whereas those injected with F5 cells were the least altered and all the other groups ranged in between.

We could not find any correlation between *in vivo* haematological parameters and other characteristics of TS/A clonal derivatives, such as *in vitro* doubling time, cell dimensions or *in vivo* tumour volume. We are currently examining three different hypotheses: either our cell lines produce a second factor which cannot be detected *in vitro*, or *in vitro* growth pattern of F clones does not allow a high CSF production, or some interaction with host environment occurs that alters CSF *in vivo* production.

When the possibility of a correlation between CSF production and metastases is considered, *in vitro* production of CSF clearly correlates with the relative metastatic capacity of E and F clones, but again *in vivo* haematologic parameters do not. Moreover, it should be borne in mind that E and F clones probably differ in the early steps of the metastatic process, since they do not give rise to significantly different numbers of lung colonies, when injected intravenously.

In conclusion, we believe that the relationship between CSF production and the metastatic process should be further explored, in particular in relation to late events (such as survival in the blood stream and attachment and growth in target organs) and possible reciprocal interactions between CSF-producing tumour cells and host cells elicited by CSF itself.

The authors wish to thank Mr A. Lorenzoni for technical assistance and Dr M.R. Motta and Dr P.L. Tazzari (Istituto di Ematologia "L. e A. Seragnoli", Bologna) for cytochemical studies. G. Nicoletti is in receipt of a training grant from C.N.R., Roma, Italy; P.-L. Lollini is in receipt of a PhD studentship (Dottorato di Ricerca in Oncologia) from M.P.I., Italy.

This work has been supported by C.N.R. Finalized Project "Oncologia", Roma, Italy (grant no. 84.00696.44), and by Associazione Italiana per la Ricerca sul Cancro, Milano, Italy.

### References

ASANO, S., URABE, A., OKABE, T. & 6 others. (1977). Demonstration of granulopoietic factor(s) in the plasma of nude mice transplanted with a human lung cancer and in the tumor tissue. *Blood*, **49**, 845.

BALDUCCI, L. & HARDY, C. (1983). High proliferation of granulocyte-macrophage progenitors in tumor-bearing mice. *Cancer Res.*, **43**, 4643.

BURLINGTON, H., CRONKITE, E.P., LAISSUE, J.A., REINCKE, U. & SHADDUCK, R.K. (1977). Colony-stimulating activity in cultures of granulocytosis-inducing tumor, *Proc. Soc. Exp. Biol. Med.*, **154**, 86.

KONWALINKA, G., GEISSLER, D., PESCHEL, CH. & 5 others. (1982). A micro agar culture system for cloning human erythropoietic progenitors *in vitro*. *Exp. Hematol.*, **10**. 71.

LEE, M.Y., SPERLIN, A. & DALE, D.C. (1980). Distribution of granulocytopoietic committed stem cells in mice with tumor induced neutrophilia. *Exp. Hematol.*, **8**, 249.

LOLLINI, P.-L., DE GIOVANNI, C., EUSEBI, V., NICOLETTI, G., PRODI, G. & NANNI, P. (1984). High-metastatic clones selected *in vitro* from a recent spontaneous BALB/c mammary adenocarcinoma cell line. *Clin. Exp. Metast.*, **2**, 251.

MILAS, L., FAYKUS, M.H., McBRIDE, W.H., HUNTER, N. & PETERS, L.J. (1984). Concomitant development of granulocytosis and enhancement of metastases formation in tumor-bearing mice. *Clin. Exp. Metast.*, **2**, 181.

MIZOGUCHI, H., SUDA, T., MIURA, Y., KUBOTA, K. & TAKAKU, F. (1982). Hemopoietic stem cells in nude mice transplanted with colony-stimulating-factor-producing tumors. *Exp. Hematol.*, **10**, 874.

NANNI, P., DE GIOVANNI, C., LOLLINI, P.-L., NICOLETTI, G. & PRODI, G. (1983). TS/A: A new metastasizing cell line from a BALB/c spontaneous mammary adenocarcinoma. *Clin. Exp. Metast.*, **1**, 373.

OKABE, T., SATO, N., KONDO, Y. & 4 others. (1978). Establishment and characterization of a human cancer cell line that produces human colony-stimulating factor. *Cancer Res.*, **38**, 3910.

OKABE, T., NOMURA, H. & OSHAWA, N. (1982). Establishment and characterization of a human colony-stimulating factor-producing cell line from a squamous cell carcinoma of the thyroid gland. *J. Natl Cancer Inst.*, **69**, 1235.

OKABE, T., FUJISAWA, M., KUDO, H., HONMA, H., OSHAWA, N. & TAKAKU, F. (1984). Establishment of a human colony-stimulating-factor-producing cell line from an undifferentiated large cell carcinoma of the lung. *Cancer*, **54**, 1024.

PESSINA, A., ERIDANI, S., BRAMBILLA, P., CATTORETTI, G., MAROCCHI, A. & MOCARELLI, P. (1981). Effect of tumor-related factors on the *in vivo* and *in vitro* colony-forming ability of normal mouse marrow cells. *Neoplasma*, **28**, 541.

PRYSTOWSKY, M.B., NAUJOKAS, M.F., HILE, J.N., GOLDWASSER, E. & FITCH, F.W. (1984). A microassay for colony-stimulating factor based on thymidine incorporation. *Am. J. Pathol.*, **114**, 149.

REINCKE, U., BURLINGTON, H., CARSTEN, A.L., CRONKITE, E.P. & LAISSUE, J.A. (1978). Hemopoietic effects in mice of a transplanted, granulocytosis-inducing tumor. *Exp. Hematol.*, **6**, 421.

SATO, N., ASANO, S., UEYAMA, Y. & 5 others. (1979). Granulocytosis and colony-stimulating activity (CSA) produced by a human squamous cell carcinoma. *Cancer*, **43**, 605.

SATO, N., MICHAELIDES, M.C. & WALLACK, M.K. (1981). Characterization of tumorigenicity, mortality, metastasis, and splenomegaly of two cultured murine colon lines. *Cancer Res.*, **41**, 2267.

SUDA, T., MIURA, Y., MIZOGUCHI, H., KUBOTA, K. & TAKAKU, F. (1980). A case of lung cancer associated with granulocytosis and production of colony-stimulating activity by the tumour. *Br. J. Cancer*, **41**, 980.

TAKEDA, A., SUZUMORI, K., SUGIMOTO, Y. & 4 others. (1984). Clear cell carcinoma of the ovary with colony-stimulating factor production. Occurrence of marked granulocytosis in a patient and nude mice. *Cancer*, **54**, 1019.


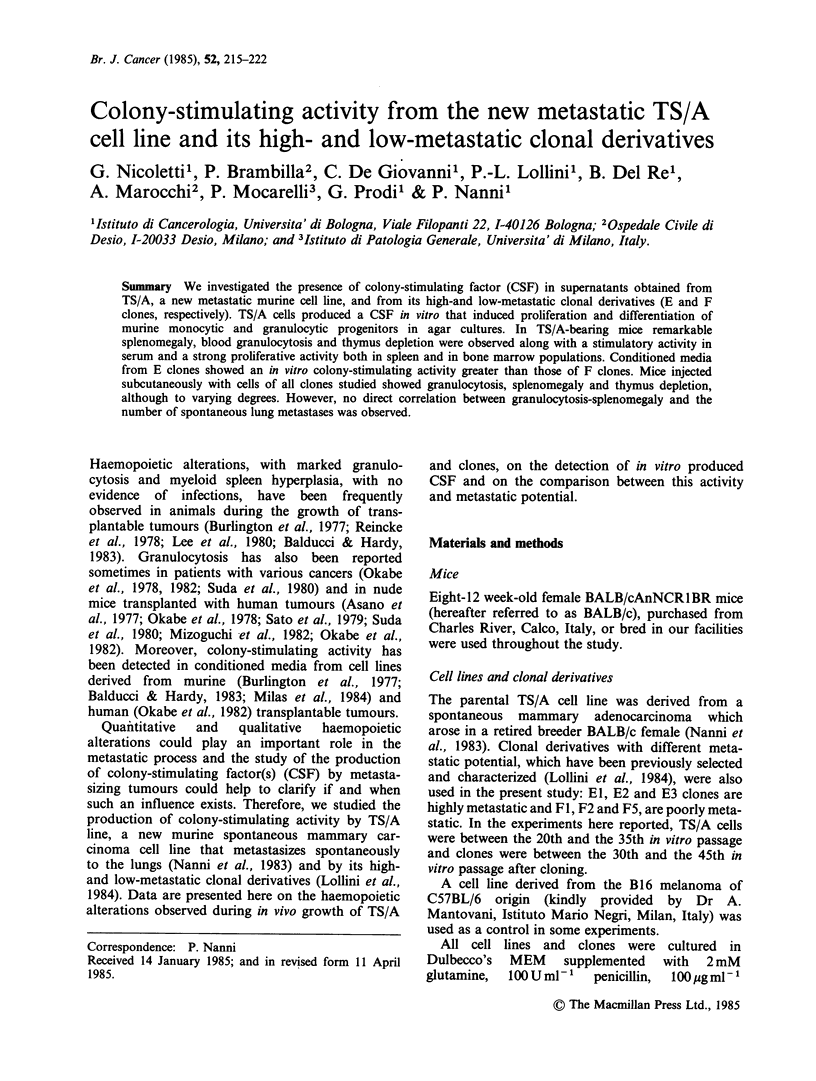

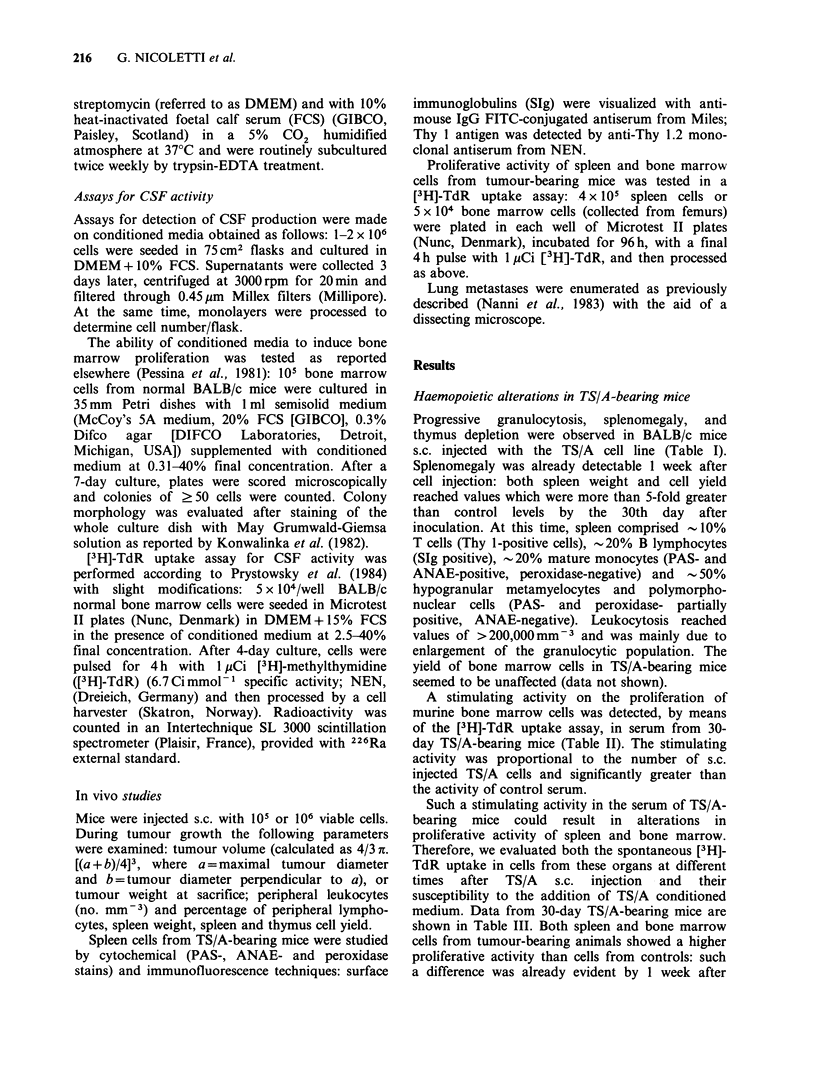

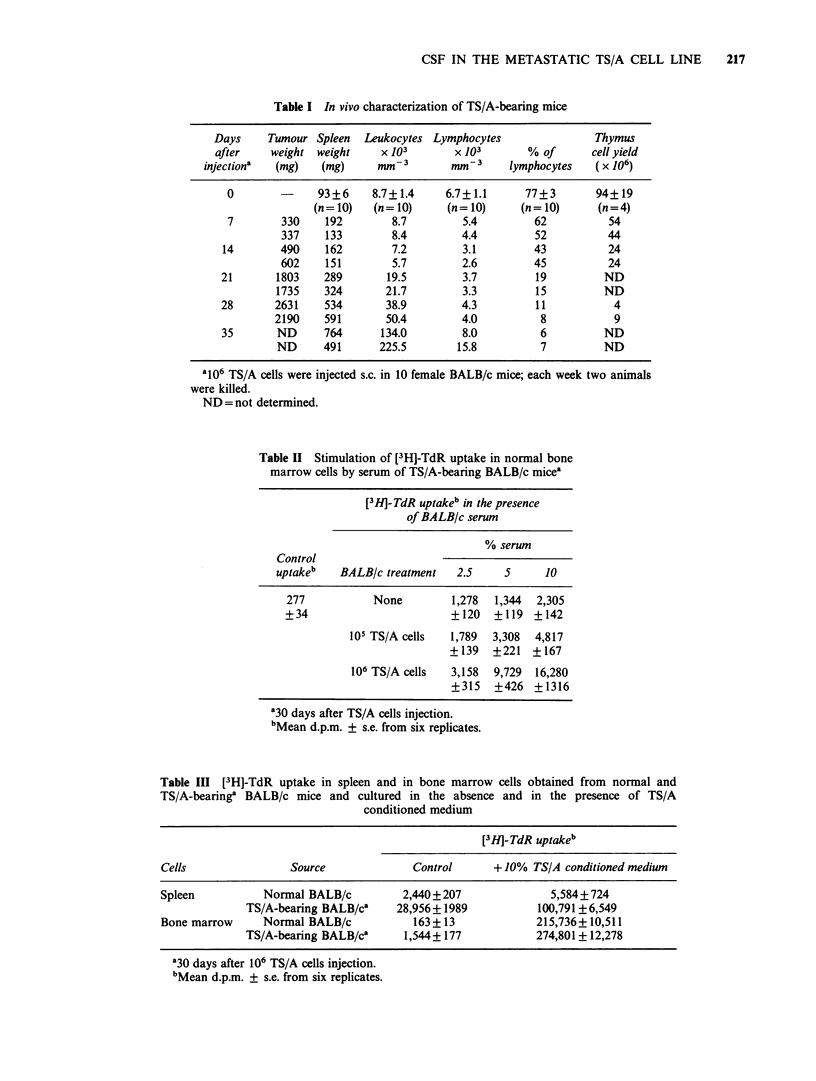

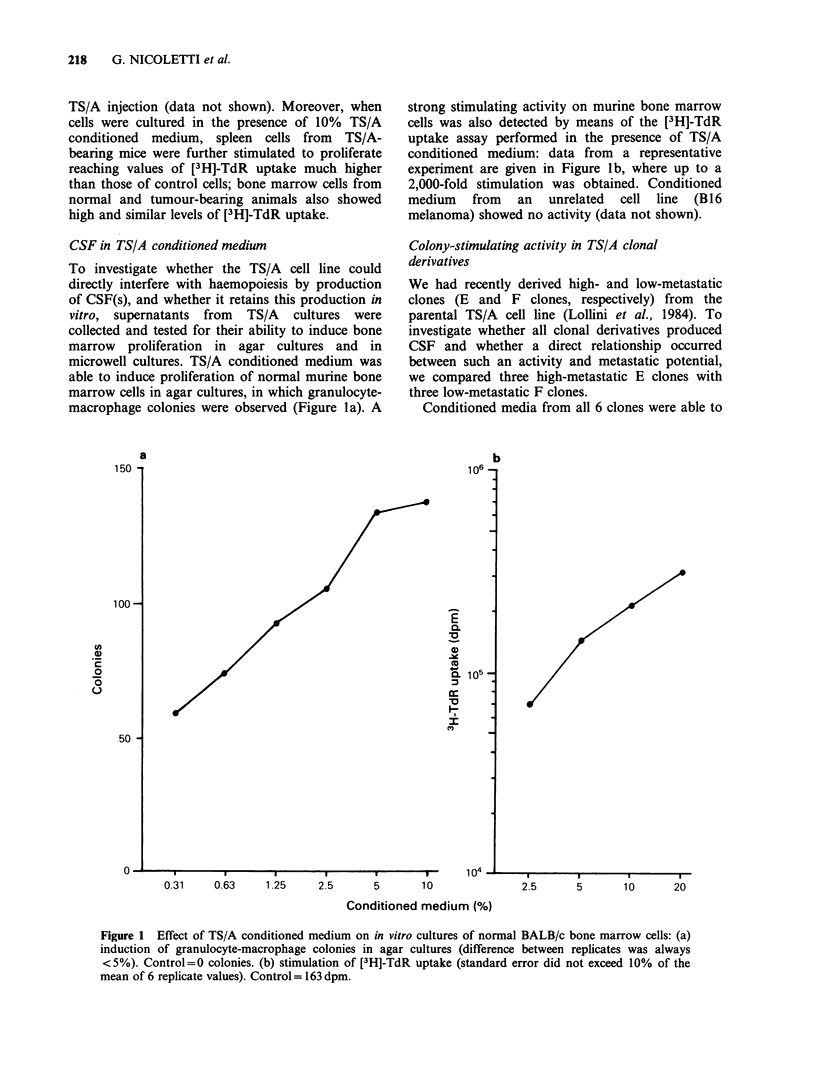

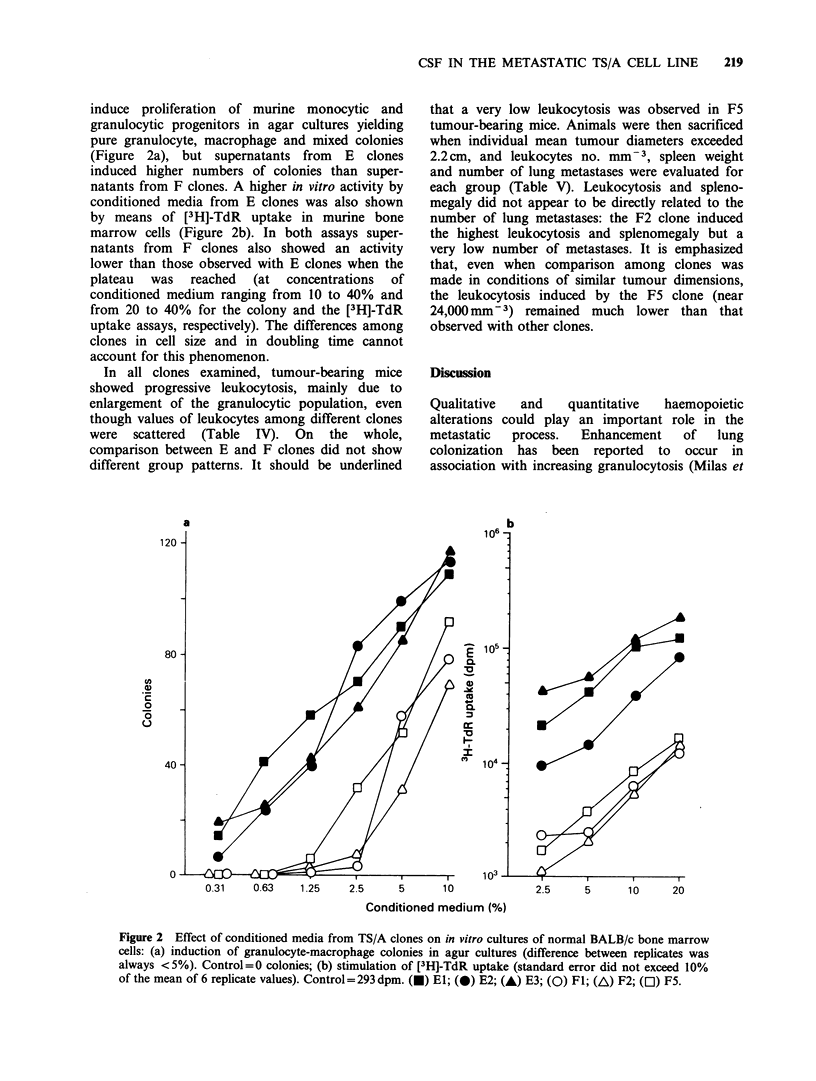

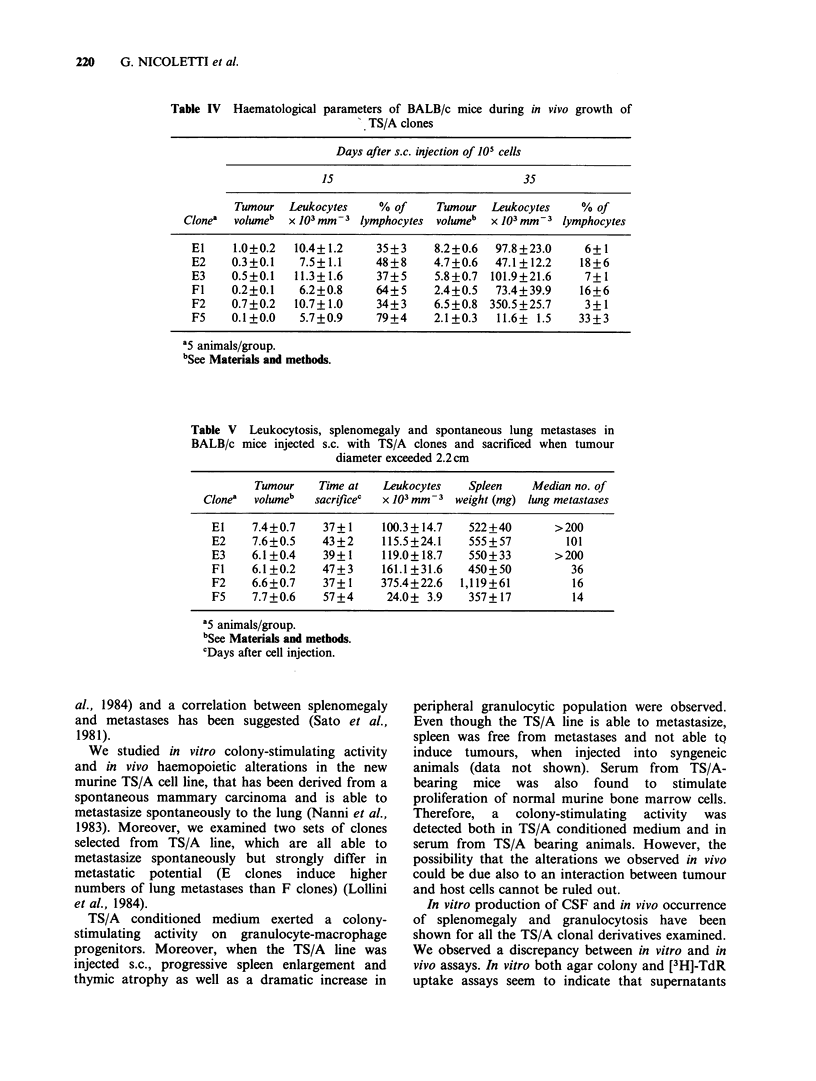

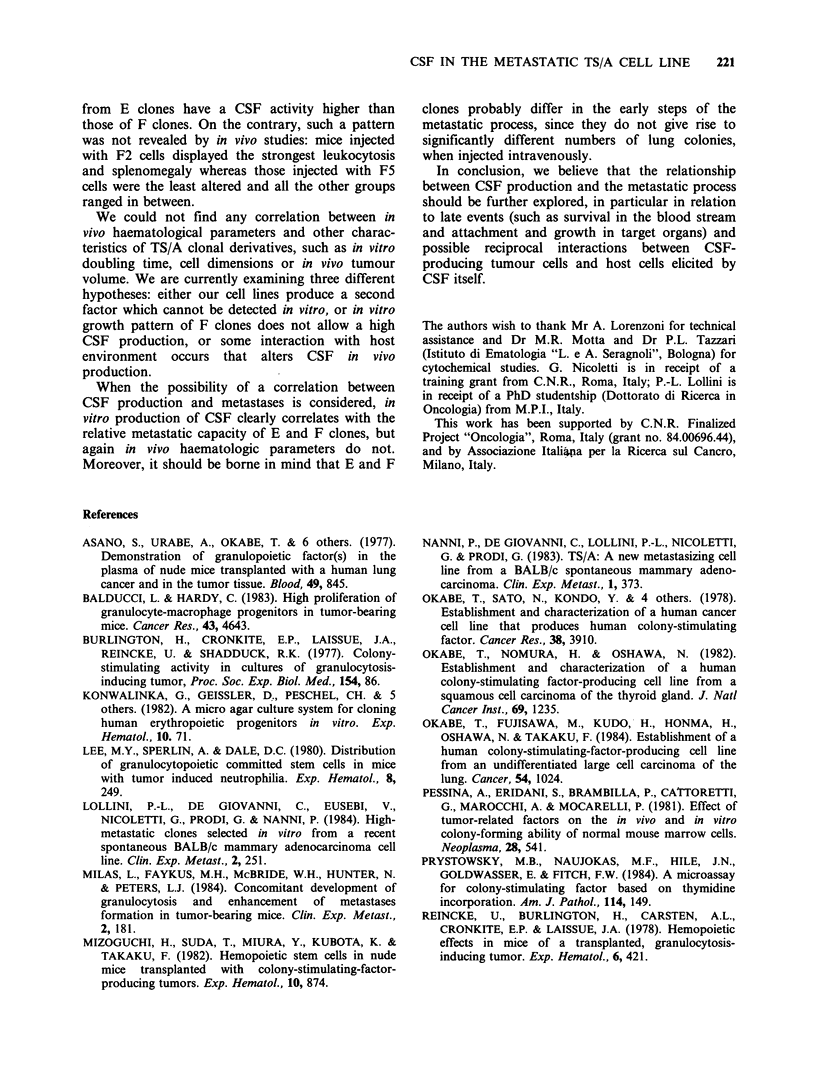

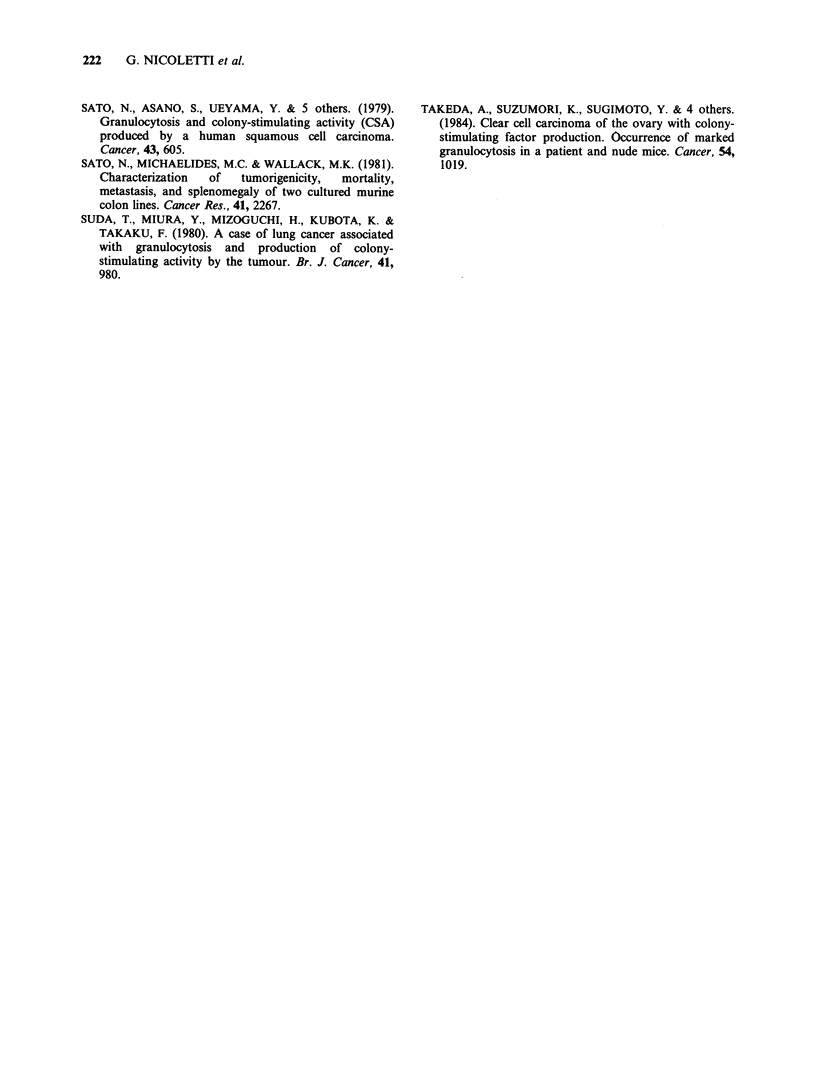


## References

[OCR_00622] Asano S., Urabe A., Okabe T., Sato N., Kondo Y. (1977). Demonstration of granulopoietic factor(s) in the plasma of nude mice transplanted with a human lung cancer and in the tumor tissue.. Blood.

[OCR_00628] Balducci L., Hardy C. (1983). High proliferation of granulocyte-macrophage progenitors in tumor-bearing mice.. Cancer Res.

[OCR_00633] Burlington H., Cronkite E. P., Laissue J. A., Reincke U., Shadduck R. K. (1977). Colony-stimulating activity in cultures of granulocytosis-inducing tumor.. Proc Soc Exp Biol Med.

[OCR_00645] Lee M. Y., Sperlin A., Dale D. C. (1980). Distribution of granulocytopoietic committed stem cells in mice with tumor induced neutrophilia.. Exp Hematol.

[OCR_00651] Lollini P. L., de Giovanni C., Eusebi V., Nicoletti G., Prodi G., Nanni P. (1984). High-metastatic clones selected in vitro from a recent spontaneous BALB/c mammary adenocarcinoma cell line.. Clin Exp Metastasis.

[OCR_00658] Milas L., Faykus M. H., McBride W. H., Hunter N., Peters L. J. (1984). Concomitant development of granulocytosis and enhancement of metastases formation in tumor-bearing mice.. Clin Exp Metastasis.

[OCR_00665] Mizoguchi H., Suda T., Miura Y., Kubota K., Takaku F. (1982). Hemopoietic stem cells in nude mice transplanted with colony-stimulating-factor-producing tumors.. Exp Hematol.

[OCR_00671] Nanni P., de Giovanni C., Lollini P. L., Nicoletti G., Prodi G. (1983). TS/A: a new metastasizing cell line from a BALB/c spontaneous mammary adenocarcinoma.. Clin Exp Metastasis.

[OCR_00690] Okabe T., Fujisawa M., Kudo H., Honma H., Ohsawa N., Takaku F. (1984). Establishment of a human colony-stimulating-factor-producing cell line from an undifferentiated large cell carcinoma of the lung.. Cancer.

[OCR_00683] Okabe T., Nomura H., Oshawa N. (1982). Establishment and characterization of a human colony-stimulating factor-producing cell line from a squamous cell carcinoma of the thyroid gland.. J Natl Cancer Inst.

[OCR_00677] Okabe T., Sato N., Kondo Y., Asano S., Ohsawa N., Kosaka K., Ueyama Y. (1978). Establishment and characterization of a human cancer cell line that produces human colony-stimulating factor.. Cancer Res.

[OCR_00697] Pessina A., Eridani S., Brambilla P., Cattoretti G., Marocchi A., Mocarelli P. (1981). Effect of tumor-related factors on the in vivo and in vitro colony-forming ability of normal mouse marrow cells.. Neoplasma.

[OCR_00704] Prystowsky M. B., Naujokas M. F., Ihle J. N., Goldwasser E., Fitch F. W. (1984). A microassay for colony-stimulating factor based on thymidine incorporation.. Am J Pathol.

[OCR_00710] Reincke U., Burlington H., Carsten A. L., Cronkite E. P., Laissue J. A. (1978). Hemopoietic effects in mice of a transplanted, granulocytosis-inducing tumor.. Exp Hematol.

[OCR_00718] Sato N., Asano S., Ueyama Y., Mori M., Okabe T., Kondo Y., Ohsawa N., Kosaka K. (1979). Granulocytosis and colony-stimulating activity (CSA) produced by a human squamous cell carcinoma.. Cancer.

[OCR_00724] Sato N., Michaelides M. C., Wallack M. K. (1981). Characterization of tumorigenicity, mortality, metastasis, and splenomegaly of two cultured murine colon lines.. Cancer Res.

[OCR_00730] Suda T., Miura Y., Mizoguchi H., Kubota K., Takaku F. (1980). A case of lung cancer associated with granulocytosis and production of colony-stimulating activity by the tumour.. Br J Cancer.

[OCR_00737] Takeda A., Suzumori K., Sugimoto Y., Yagami Y., Miyazawa T., Yamada C., Matsuyama M. (1984). Clear cell carcinoma of the ovary with colony-stimulating-factor production. Occurrence of marked granulocytosis in a patient and nude mice.. Cancer.

